# Enhanced Expression of ANO1 in Head and Neck Squamous Cell Carcinoma Causes Cell Migration and Correlates with Poor Prognosis

**DOI:** 10.1371/journal.pone.0043265

**Published:** 2012-08-17

**Authors:** Christian Ruiz, Joana Raquel Martins, Florian Rudin, Sandra Schneider, Tanja Dietsche, Claude A. Fischer, Luigi Tornillo, Luigi M. Terracciano, Rainer Schreiber, Lukas Bubendorf, Karl Kunzelmann

**Affiliations:** 1 Institute for Pathology, University Hospital Basel, Basel, Switzerland; 2 Department of Otolaryngology, Head and Neck Surgery, Kantonsspital Graubünden, Chur, Switzerland; 3 Institut für Physiologie, Universität Regensburg, Regensburg, Germany; The Chinese University of Hong Kong, Hong Kong

## Abstract

Head and neck squamous cell carcinoma (HNSCC) has the potential for early metastasis and is associated with poor survival. Ano1 (Dog1) is an established and sensitive marker for the diagnosis of gastrointestinal stromal tumors (GIST) and has recently been identified as a Ca^2+^ activated Cl^−^ channel. Although the *ANO1* gene is located on the 11q13 locus, a region which is known to be amplified in different types of human carcinomas, a detailed analysis of Ano1 amplification and expression in HNSCC has not been performed. It is thus still unclear how Ano1 contributes to malignancy in HNSCC. We analyzed genomic amplification of the 11q13 locus and Ano1 together with Ano1-protein expression in a large collection of HNSCC samples. We detected a highly significant correlation between amplification and expression of Ano1 and showed that HNSCC patients with Ano1 protein expression have a poor overall survival. We further analyzed the expression of the Ano1 protein in more than 4′000 human samples from 80 different tumor types and 76 normal tissue types and detected that besides HNSCC and GISTs, Ano1 was rarely expressed in other tumor samples or healthy human tissues. In HNSCC cell lines, expression of Ano1 caused Ca^2+^ activated Cl^−^ currents, which induced cell motility and cell migration in wound healing and in real time migration assays, respectively. In contrast, knockdown of Ano1 did not affect intracellular Ca^2+^ signaling and surprisingly did not reduce cell proliferation in BHY cells. Further, expression and activity of Ano1 strongly correlated with the ability of HNSCC cells to regulate their volume. Thus, poor survival in HNSCC patients is correlated with the presence of Ano1. Our results further suggest that Ano1 facilitates regulation of the cell volume and causes cell migration, which both can contribute to metastatic progression in HNSCC.

## Introduction

ANO1 is also known as TMEM16A (anoctamin 1, DOG1, ORAOV2, TAOS2, FLJ10261) and is an established biomarker for gastrointestinal stromal tumors (GISTs) [1], [2]. The coding sequence of Ano1 is located within the 11q13 region, a chromosomal locus that is frequently amplified in a number of different human cancers, such as urinary bladder cancer, breast cancer and head and neck squamous cell carcinoma (HNSCC) [3]. The complex structure of this amplicon has been studied mostly in breast cancer, which shows overexpression of a number of driver genes [4], [5]. In oral squamous cell carcinoma, the 11q13 amplicon has also been reported as a prevalent finding [6]. Recently, enhanced expression of the *Ano1* gene in HNSCC has been associated with the propensity for distance metastasis [7].

Ano1 appears to be sparsely expressed in the human body. It was found in the apical membrane of epithelial cells of airways and gastrointestinal tract. It has also been detected as a selective marker in Cajal pacemaker cells [8]. Notably Ano1 has been identified recently as a Ca^2+^ activated Cl^−^ channel [9]–[11]. There is a well recognized link between expression of ion channels, cell proliferation and development of cancer [12]–[14]. As intracellular Ca^2+^ fluctuations are a hallmark of both cell proliferation and migration, Ca^2+^ activated Cl^−^ channels and thus Ano1 have also been found to have a role during cell cycling and migration [7], [13], [15]–[18].

**Figure 1:**
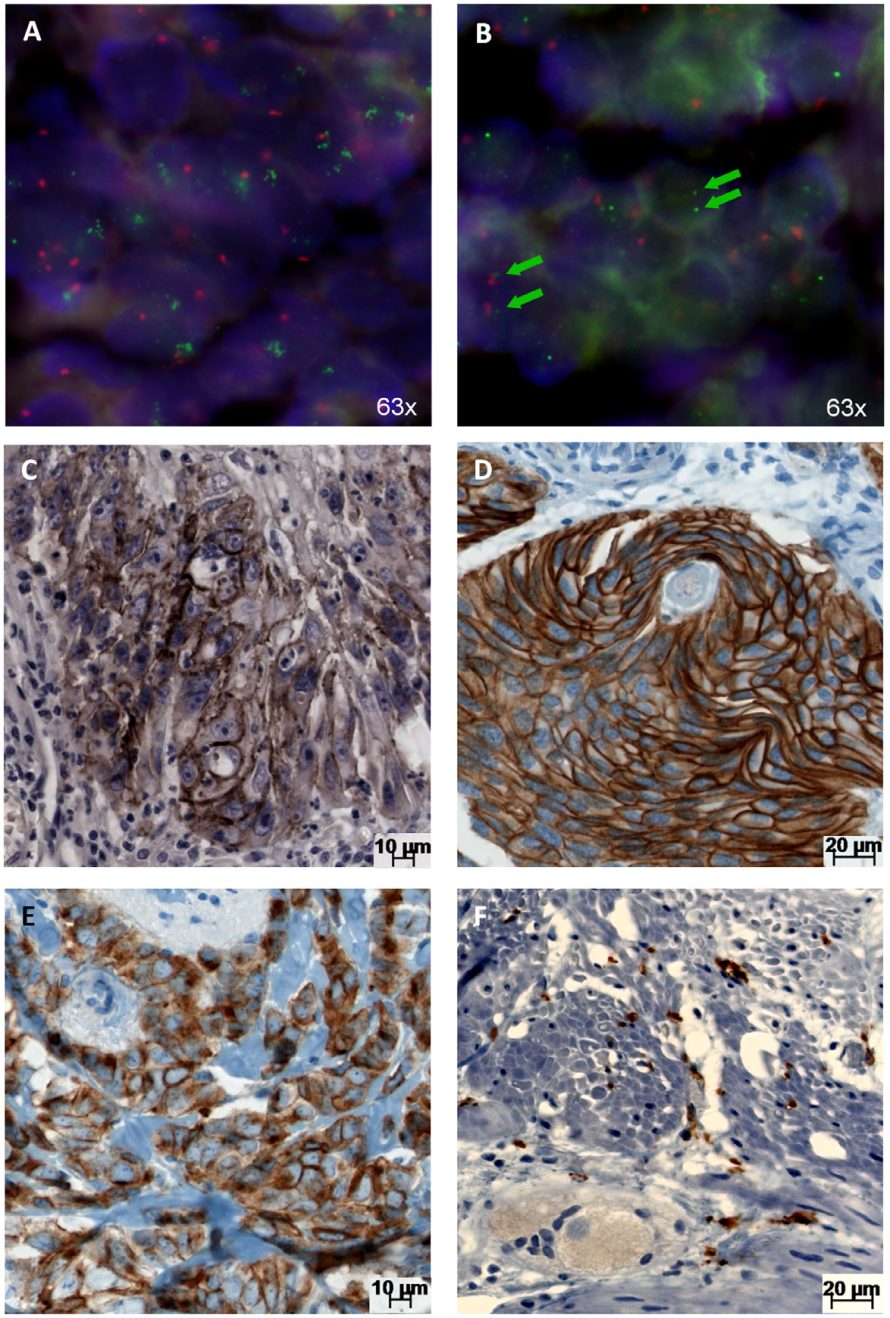
Typical images of Ano1 gene amplification and protein expression in human tissues. A–B) FISH analysis of *Ano1* amplified (A) and non-amplified (B) HNSCC from the oral cavity. C–E) Immunohistochemical analysis of Ano1 expression in different tissue types. C) HNSCC of the oral cavity: strong membranous staining of the tumor cells. D) Invasive urothelial bladder cancer: strong membranous staining of the tumor cells. E) Invasive lobular breast cancer: cytoplasmic and membranous staining of the tumor cells. F) Normal muscular wall of the appendix: Ano1 protein positivity was detected in the interstitial cells of Cajal. Green signals: *Ano1* gene. Red signals: Centromere 11. Green arrows point towards Ano1 genes.

A recent report showed that overexpression of Ano1 in HNSCC does not lead to increased proliferation, while in another study convincing evidence was provided for control of proliferation through ERK1/2 activation and induction of cyclin D1 by Ano1 [7], [18]. It was also suggested that Ano1 amplification and expression is a marker for distant metastasis in HNSCC. Ano1 was proposed to control cell properties that are important for metastasis. Cell migration is a crucial property in both physiological and patho-physiological processes, including wound healing and tumor metastasis. In fact previous studies associated altered cell migration with tumor malignancy and metastasis [19], [20]. In the present study we examined genomic amplification and expression of Ano1 in a large number of human HNSCC samples. The goal was to identify the functional consequences of Ano1 expression in HNSCC cells. We found that Ano1 strongly supports the ability of HNSCC cells to migrate and that migration is correlated to the ability to regulate cell volume.

**Table 1:** Analysis of the 11q13 (Ano1 and CCND1) genomic amplification and protein expression in 365 HNSCCs.

A									
		FISH (*ANO1*)				IHC (Ano1)			
Tumor location	n on TMA	Evaluable (n)	Amplified	Non-amplified	Percentage	Evaluable (n)	Positive	Negative	Percentage
Oral cavity	129	96	6	90	6.3	103	5	98	4.9
Oropharynx	110	77	13	64	16.9	71	3	68	4.2
Larynx	76	39	4	35	10.3	31	4	27	12.9
Hypopharynx	50	30	17	13	56.7	37	7	30	18.9
***Total:***	*365*	*242*	*40*	*202*	*16.5*	*242*	*19*	*223*	*7.8*
**B**									
			**IHC (Ano1)**						
	**n**	**Category**	**Positive**	**Negative**	**P-value**				
**FISH (** ***ANO1*** **)**	156	Non-amplified	5 (33%)	151 (88%)	<0.0001				
	31	Amplified	10 (67%)	21 (12%)					
**C**									
			***CCND1*** ** (FISH)**						
	**n**	**Category**	**Amplified**	**Non-amplified**	**P-value**				
***ANO1*** ** (FISH)**	31	Amplified	30 (97%)	1 (3%)	<0.0001				
	156	Non-amplified	2 (1%)	172 (99%)					

**Table 1** **Legend.** A) Overview of the prevalence of genomic amplification of *Ano1* and Ano1 expression in HNSCCs from different tumor locations.

B)Genomic amplification of the 11q13 locus is associated with the presence of Ano1 protein expression.

C)Strong correlation between the genomic amplification of *Ano1* and *CCND1*.

## Results

### Genomic Amplification and Protein Expression of Ano1 in HNSCC

In order to determine the clinical significance of *Ano1* gene amplification and Ano1 protein expression in HNSCC, we applied fluorescence in-situ hybridization (FISH) and immunohistochemistry (IHC) to a defined set of 365 primary HNSCCs with clinicopathological and follow-up data [21]. Representative images are shown in Figure 1. We detected genomic amplification of the *Ano1* gene locus in 16.5% of the HNSCCs. Notably; the distribution amongst HNSCCs from distinct sites was heterogeneous: the prevalence of *Ano1* gene amplification ranged from 6% in tumors from the oral cavity up to 57% in SCCs of the hypopharynx (Table 1A). A similar distribution was observed for Ano1 protein: Ano1 expression was detected in 4–5% of the SCCs from oral cavity and oropharynx, but in 19% of tumors from hypopharynx. Despite the lower incidence of Ano1-protein expression (8%) in comparison to the amplification (16.5%), there was a strong correlation between these two parameters (p<0.0001, Table 1B): two thirds of the Ano1 expressing HNSCCs had a genomic amplification of the *Ano1* gene. We further analyzed the genomic status of *CCND1*, which is also located on 11q13, and observed a co-amplification of these two genes in almost all of the cases (p<0.0001, Table 1C). These findings strongly suggest the genomic amplification of the 11q13 locus as one of the main mechanisms for the expression of Ano1 in HNSCC.

**Figure 2:**
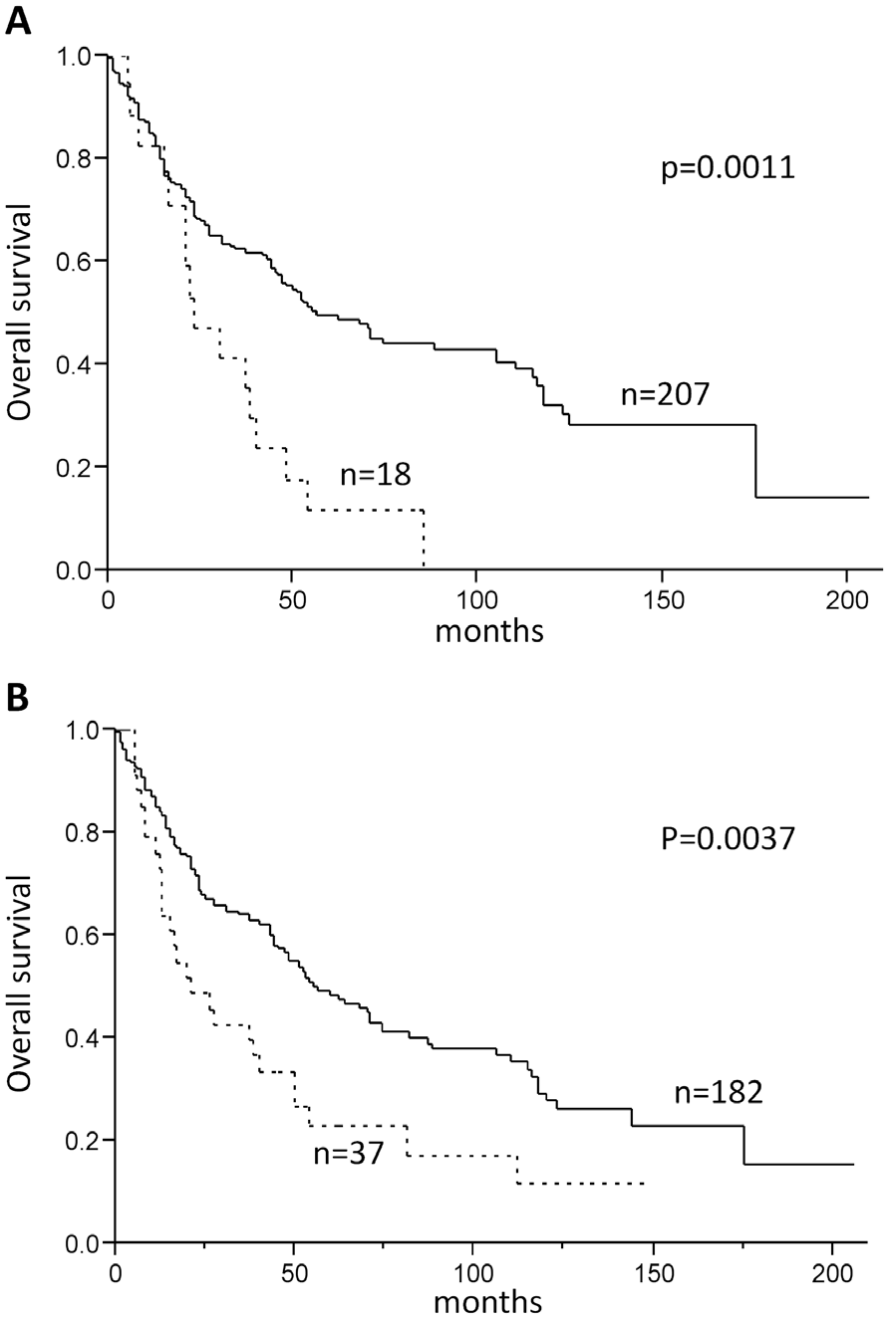
Survival analysis of patients with HNSCC. A) Kaplan-Meier curve analysis illustrating a worse overall survival for HNSCC patients with detectable Ano1 protein expression (solid line: Ano1 protein negative, dashed line: Ano1 protein positive). B) Kaplan-Meier curve analysis demonstrating a worse outcome for patients with 11q13 (*Ano1)* amplified HNSCC. (Solid line: normal *Ano1* gene copy status, dashed line: 11q13 amplified).

### Role of Ano1 for Survival in Patients with HNSCC

Next we investigated the impact of Ano1 on survival. Both, genomic amplification and protein expression of Ano1 were strong predictors of poor outcome (Figure 2). The median survival time for patients with Ano1-positive tumors was 23 months (95% CI, 15–40) compared to 56 months (95% CI, 46–105) for Ano1-negative cases (p = 0.0011, Figure 2A). Similar was observed for *Ano1* amplification: patients with *Ano1*-amplification showed a median overall survival of 21 months (95% CI, 13–40), whereas patients without *Ano1*-amplification showed a median overall survival of 55 months (95% CI, 45–75) (p = 0.0037, Figure 2B). Multivariate analysis including tumor stage (pT) and tumor location (both significant in univariate analysis) revealed Ano1 positivity (p = 0.022), but not *Ano1* gene amplification (p = 0.382) as independent prognostic factor besides pT stage (p = 0.0003) (Table S3). These findings indicate that Ano1 positivity identifies a subgroup of HNSCC with adverse prognosis.

**Table 2:** Comprehensive analysis of Ano1 protein expression in human tumors.

			Ano1 (IHC)			
Organ	Tumor type	Spots on TMA (n)	Analyzable (n)	Negative (n)	Positive (n)	Positivity (%)
Brain	Glioblastoma multiforme	44	38	37	1	3
Endometrium	Endometroid carcinoma	49	47	43	4	9
Esophagus	Adenocarcinoma	9	8	7	1	13
	Squamous cell carcinoma	33	31	27	4	13
Gall bladder	Adenocarcinoma	45	37	33	4	11
	Normal	9	6	3	3	50
Larynx	Squamous cell carcinoma	58	38	34	4	11
Liver	Hepatocellular carcinoma	97	81	79	2	2
Oral cavity	Squamous cell carcinoma	50	46	40	6	13
Pancreas	Adenocarcinoma	49	42	39	3	7
Salivary gland	Warthin tumors	25	18	18*	0	0
	Pleomorphic adenoma	49	34	33	1	3
	Adenoidcystic carcinoma	33	28	27	1	4
Small intestine	Adenocarcinoma	22	19	18	1	5
Soft tissue	Leiomyosarcoma	35	35	33	2	6
	GIST	12	11	0	11	100
	Normal (sceletal muscle)	24	18	15	3	17
Urinary bladder	TCC non-invasive (pTa)	38	36	35	1	3
	TCC invasive (pT2-4)	78	60	59	1	2

* These tumors only showed unspecific cytoplasmic Ano1 staining and were thus classified as negative.

**Table 2** **Legend.** Summary of human tumors with detectable Ano1 protein expression. Most tumors do not express Ano1 (see Table S4 for complete listing).

### Analysis of Ano1 Protein Expression Across 80 Different Tumor Types and 76 Normal Tissue Types

In order to analyze the distribution of the Ano1 protein expression in tumors other than HNSCC, we applied Ano1 immunohistochemistry to a multi tumor TMA composed of 3417 tissue samples (including 315 normal controls) of 80 different tumor types. Only 53 out of 2837 tissue specimens expressed Ano1, predominantly in the cell membrane (Table 2, Table S4). Ano1 expression was more frequent (>10%) in HNSCC, GISTs (100%), adenocarcinomas and squamous cell carcinomas of the esophagus (both 13%) and adenocarcinomas of the gall bladder (11%). We also analyzed another TMA comprising 608 normal samples from 76 different tissue types [22]. Ano1 was only expressed in the epithelium of gall bladder, stomach and duodenum (Table 3A, Table S5). Moreover, Ano1 was detected in the apical membrane of serous glands and interstitial cells of Cajal in several organs (Table 3B–C, Figure 1F). Taken together, most normal human tissues and tumors do not express Ano1.

**Table 3:** Comprehensive analysis of Ano1 protein expression in normal tissues.

A		
*Positivity:*	Ano1 (IHC)	
Organ	Evaluable (n)	Positive (n)
Gall bladder, epithelium	3	3
Stomach, antrum	4	4
Duodenum, Brunner gland	5	1
**B**		
***Apical positivity:***	**Ano1 (IHC)**	
**Organ**	**Evaluable (n)**	**Positive (n)**
Bronchial gland^#^	4	4
Small salivary gland^#^	1	1
Sublingual gland^#^	3	3
Endocervix	3	2
Endometrium	5	2
Epididymis	2	1
Fallopian tube mucosa	2	2
Parotid gland	4	4
Submandibular gland	8	8
Seminal vesicle	1	1
**C**		
***Positive cajal cells:***	**Ano1 (IHC)**	
**Organ**	**Evaluable (n)**	**Positive (n)**
Appendix, mucosa	2	1
Appendix, muscular wall	7	6
Colon descendens, muscular wall	7	4
Ileum muscularis	5	3
Stomach, muscular wall	6	6

#Mucosa and mucous glands were negative.

Summary of normal tissues with detectable Ano1 protein expression. Most normal tissues do not express Ano1. Only gall bladder, stomach and duodenum express Ano1 (A). Apical positivity (B) and Ano1-positive Cajal cells (C) were detected in several types of tissues. (See Table S5 for complete listing.).

### Correlation between 11q13-amplification and Ano1 Expression in Non-HNSCC

We examined whether Ano1 expression in non-HNSCC might be caused by genomic amplification of the 11q13 locus. To that end, we constructed a small TMA from available Ano1 protein expressing non-HNSCC tumors from the multi tumor TMA (Table S1A) and from 11q13 amplified breast and bladder cancers from previous studies (Table S1B) [23], [24]. Interestingly, none of the tumors from the multi tumor TMA (endometrium carcinoma, esophagus SCC, hepatocellular carcinoma (HCC), glioblastoma multiforme (GBM), GIST, pancreas adenocarcinoma, adenoidcystic carcinoma of the salivary gland) with Ano1 protein expression showed amplification of the 11q13 locus (Table S1A). In contrast, Ano1 was expressed in a remarkable fraction of 11q13-amplified breast (4 out of 11) and bladder cancers (6 out of 14), indicating a correlation between amplification of 11q13 and Ano1 protein in these two types of cancer (Table S1B, Figure 1D-E).

**Figure 3:**
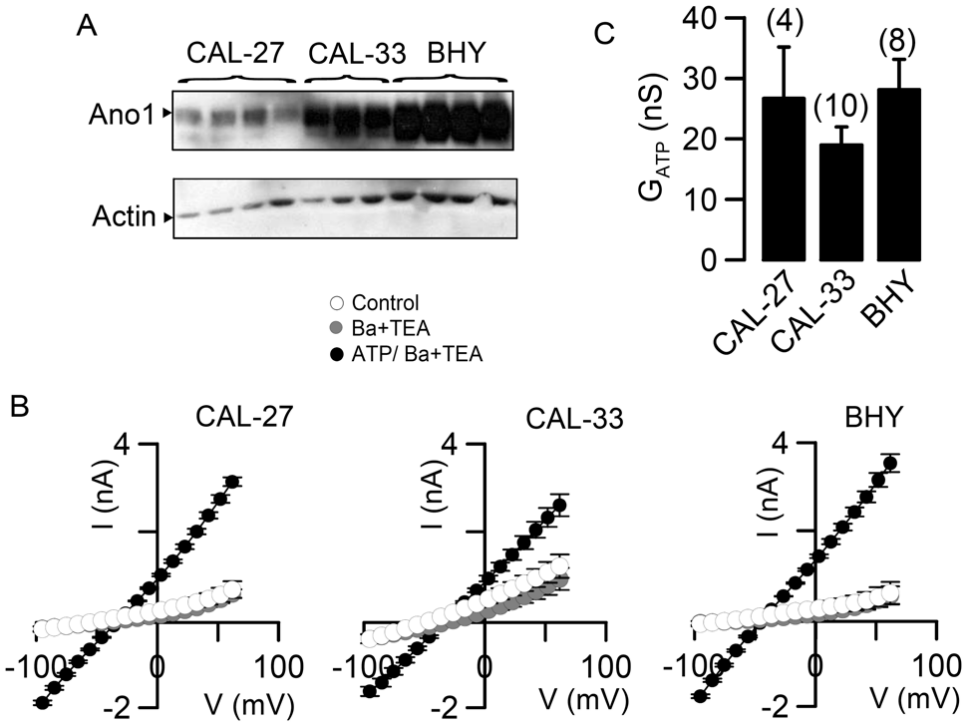
Expression of Ano1 and Ca^2+^ activated Cl^−^ currents in HNSCC cells. A) Western blots of Ano1 expressed in the three different human cell lines CAL-27, CAL-33, and BHY. B) Current/voltage relationships of whole cell currents activated by 100 µM ATP (filled circles) in CAL-27, CAL-33, and BHY cells. Application of the K^+^ channel inhibitors Ba^2+^ (5 mM) and TEA^+^ (10 mM) (grey circles) did not significantly change currents, suggesting a dominant role Cl^−^ currents. C) Summary of the calculated ATP-activated whole cell conductances. Mean ± SEM, (number of experiments).

### Expression of Ano1 and Ca^2+^ Activated Cl^−^ Currents in HNSCC Cells

We further analyzed expression of Ano1 in three different HNSCC cell lines: CAL-27 and CAL-33 (two tongue squamous cell carcinoma cell lines without genomic amplification of Ano1) and BHY cells (an oral squamous cell carcinoma cell line with known 11q13-amplification [25], [26]. Western blot analysis showed very low levels of Ano1 in CAL-27 cells. Expression levels were higher in CAL-33 cells, and reached highest levels in BHY cells, which may be explained by amplification of the 11q13 locus in BHY cells (Figure 3A). Ano1-mRNA levels in the three cells lines showed a distribution similar to that of Ano1-protein (data not shown). Ano1 forms chloride ion channel activated by intracellular Ca^2+^. This was shown again by expression of Ano1 in HEK293 cells, which caused a Ca^2+^ activated Cl^−^ conductance of 45.6±3.6 nS (n = 7). In contrast, mock transfected control cells did not significantly change their whole cell conductance upon increase in intracellular Ca^2+^ by 1 µM ionomycin (0.2±0.05 nS (n = 8)). We examined Ca^2+^ activated whole cell Cl^−^ currents these cell lines. The agonist of purinergic P2Y_2_ receptors, ATP (100 µM), is known to increase intracellular Ca^2+^ ([Ca^2+^]_i_) and to activate Ano1 [27], [28](31;41). Surprisingly ATP activated large whole cell Cl^−^ currents of similar magnitude in CAL-27, CAL-33, and BHY cells, although expression of Ano1 differed remarkably between these cell lines (Figure 3). This may be explained by the fact that only a fraction of Ano1 protein reaches the cell membrane.

**Figure 4:**
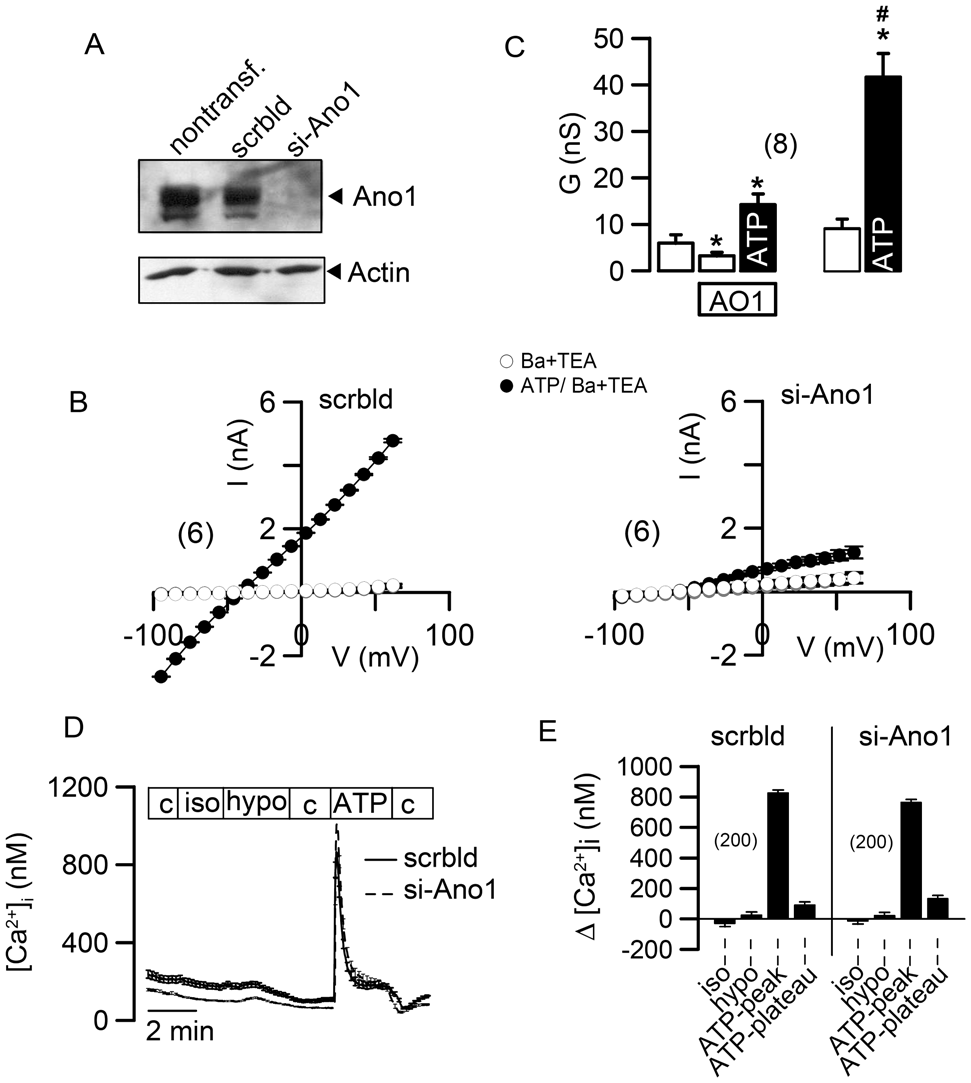
Ano1 causes Ca^2+^ dependent Cl^−^ currents and does not influence intracellular Ca^2+^ signaling. A) Western blot of Ano1 in nontransfected BHY cells, and in cells treated with scrambled RNA or siRNA-Ano1, indicating knockdown of Ano1 in siRNA-treated cells. B) Current/voltage relationships of whole cell currents in BHY cells treated with scrambled RNA (left panel) or siRNA-Ano1 (right panel). Currents are shown under control conditions (open circles) and after activation by 100 µM ATP (filled circles). C) Summary of the whole cell conductances under control conditions (white bars) and after stimulation with ATP (filled bars) in the presence or absence of the Ano1 channel inhibitor AO1 (20 µM) and ATP (100 µM). D) Recording of the intracellular Ca^2+^ concentration in BHY cells treated with scrambled RNA or siRNA-Ano1, and effects of control solution (c), isotonic solution in which 50 mM NaCl was replaced by 100 mM mannitol (iso), hypotonic solution in which 100 mM mannitol were removed (hypo), and 100 µM ATP. E) Summary of the changes in the intracellular Ca^2+^ concentration after the different maneuvers described in (D). Mean ± SEM, (number of experiments). *indicates significant difference (p<0.05, paired t-test). ^#^indicates significant difference (p<0.05, unpaired t-test).

### Ano1 causes Ca^2+^ Dependent Cl^−^ Currents but does not Influence Intracellular Ca^2+^ Signaling

In order to demonstrate that Ano1-expression is in charge of Ca^2+^ activated whole cell currents, we knocked down Ano1 by siRNA, which strongly reduced whole cell currents activated by ATP. (Figure 4A,B). We further demonstrated the role of Ano1 for Ca^2+^ dependent Cl^−^ secretion using the channel blocker AO1 (20 µM). Activation of the Ano1 whole cell conductance (left filled bar in Fig. 4C) was significantly reduced in the presence of AO1 and when compared with the conductance in the absence of AO1 (right filled bar in Fig. 4C) [28], [29]. Moreover, we excluded the possibility that Ano1-knockdown affects intracellular Ca^2+^ signaling, which would compromise activation of Ano1: ATP induced similar increase in peak and plateau [Ca^2+^]_i_ in the absence or presence of siRNA-Ano1 (Figure 4D,E). Surprisingly, when exposed to hypotonic bath solution (200 mosmol/l) BHY cells did not show an increase [Ca^2+^]_i_, which is typically observed when cells undergo hypotonic cell swelling (Figure 4D,E). This suggested an exquisite volume regulation in these cells, which was confirmed by subsequent volume measurements (see below).

**Figure 5:**
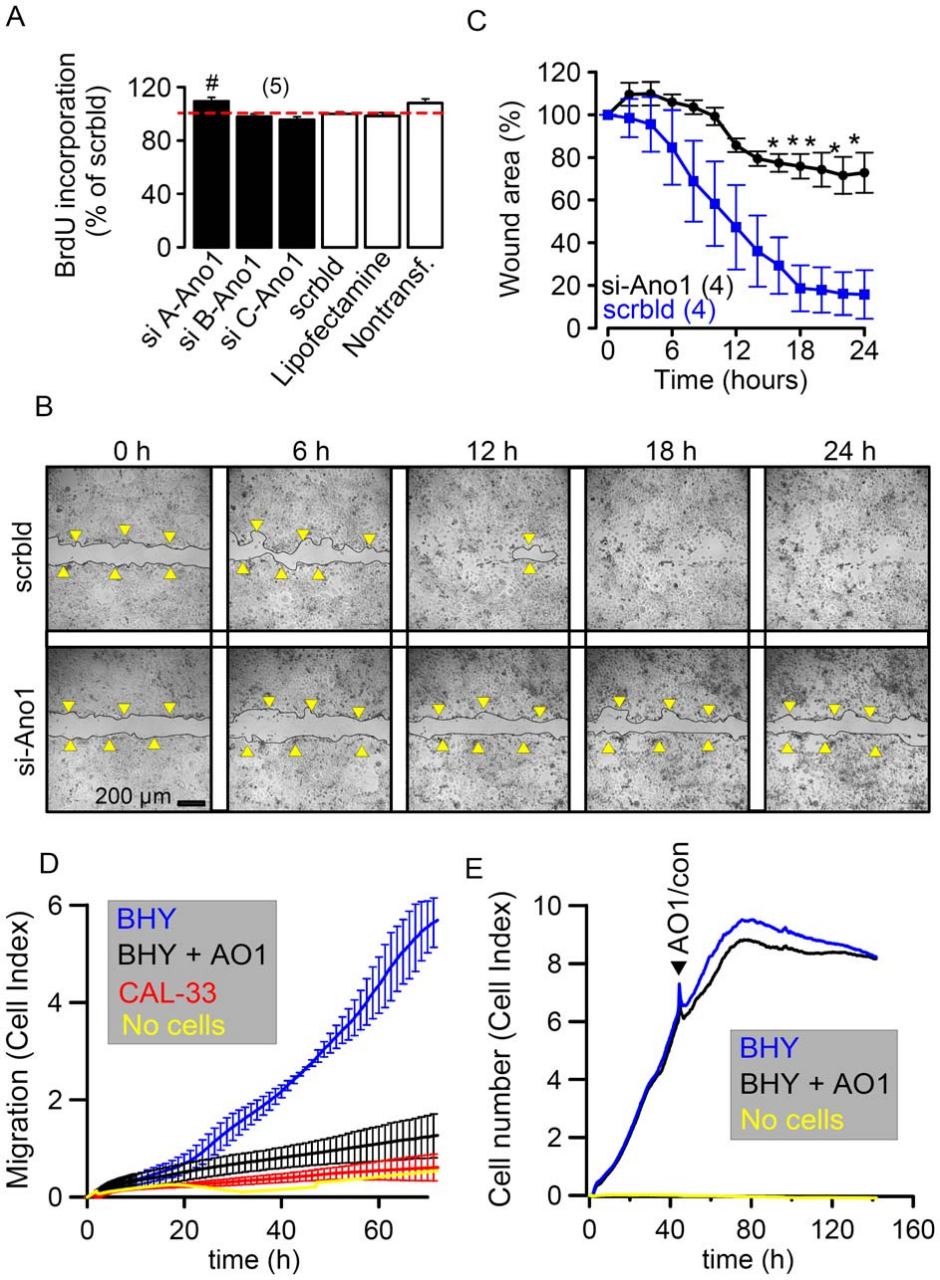
*Ano1 causes cell migration.* A) Summary of BrdU incorporation in BHY cells after various treatments, shown as % of BrdU incorporation, when compared to cells treated with scrambled RNA (red dashed line). B) Representative images of wound healing in a scratch assay with BHY cells, treated with scrambled RNA (upper panels) or after Ano1-knockdown with siRNA-Ano1 (lower panels). Yellow arrowheads indicate edges of the damage. C) Time course for the wound healing process (closure of the scratch) for control cells (scrambled RNA) and after Ano1-knockdown (si-Ano1). D) Migration of BHY and CAL-33 cells measured continuously as cell index using the xCELLigence system. Inhibition of migration by blockade of Ano1 with AO1 (10 µM). E) Cell Proliferation measured continuously as cell index using the xCELLigence system. AO1 (10 µM) has no effect on proliferation. Experiments were performed at least in triplicates. Mean ± SEM, (number of experiments). ^#^indicates significant difference (p<0.05, ANOVA).

### Ano1 Supports Cell Migration

Because the present data demonstrate a correlation between expression of Ano1 and cancer, we examined possible effects of Ano1-knockdown on proliferation of BHY cells, using three different siRNAs. To our surprise and in contrast to a previous report [18], knockdown of Ano1 did not reduce proliferation of BHY cells as shown by BrdU incorporation (Figure 5A). Proliferation of BHY cells was also measured continuously in real time using the xCELLigence system (c.f. Methods). Inhibition of Ano1 channel currents by AO1 (10 µM) did not significantly affect proliferation (Fig. 5E). Similar results were obtained in pancreatic cancer cells (CF-PAC1), which also express Ano1 [30], [31] (File S1A). Conversely, when Ano1 or a mutant form of Ano1 with reduced Cl^−^ channel activity (Ano1-K160A) [30] were overexpressed in CF-PAC1 cells, proliferation was rather reduced (File S1B). These results suggest that although Ano1 is able to induce proliferation and cancer progression in HNSCC tumor xenografts and a number of cell lines, it does not support cell proliferation in every cell type.

**Figure 6:**
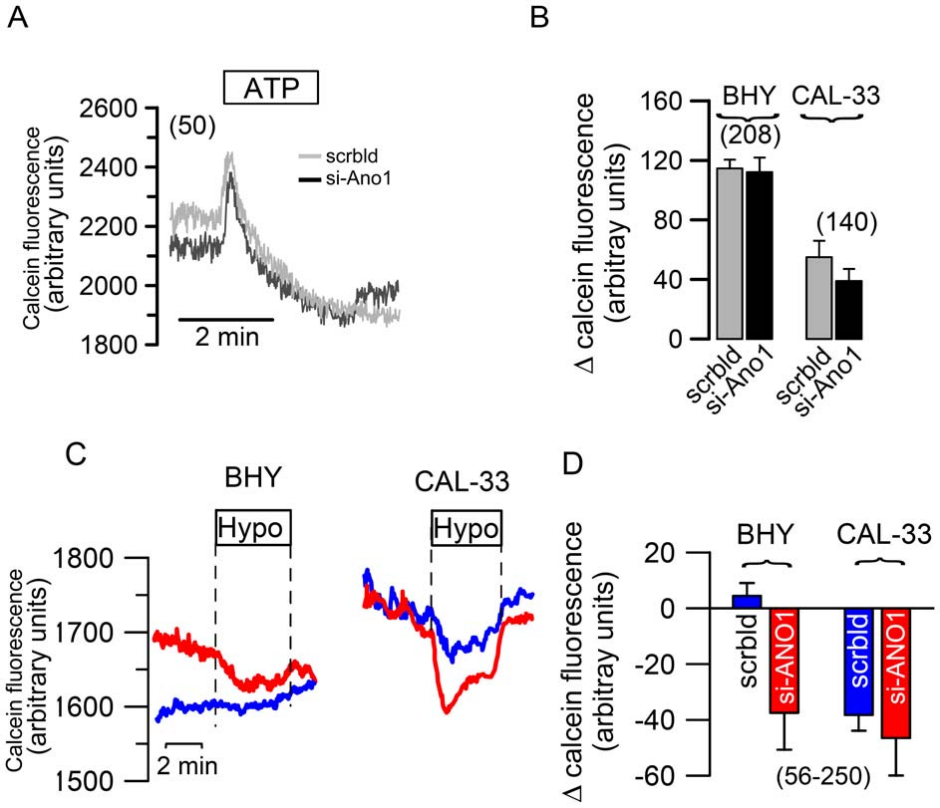
Ano1 supports regulatory volume decrease. A) Original recordings of the calcein fluorescence in BHY cells, treated with scrambled RNA or siRNA-Ano1, before and after stimulation with 100 µM ATP. ATP induces transient cell shrinkage that was not affected by siRNA-knockdown of Ano1. B) Summary of the ATP-induced changes in calcein fluorescence in BHY and CAL-33 cells treated with scrambled RNA or siRNA-Ano1. C) Original recordings of calcein fluorescence in BHY and CAL-33 cells treated with scrambled RNA (blue) or siRNA-Ano1 (red) and effect of a hypotonic (Hypo; 200 mosmol/l) bath solution. D) Summary of the hypotonicity-induced changes in calcein fluorescence in BHY and CAL-33 cells treated with scrambled RNA or siRNA-Ano1. Mean ± SEM, (number of experiments). *indicates significant difference (p<0.05, paired t-test). ^#^indicates significant difference (p<0.05, paired t-test).

We further examined a possible role of Ano1 for volume regulation and cell migration, which is crucial for metastasis and enhanced malignancy [7], [32]. Confluent BHY monolayers were wounded with a sterile pipette tip (scratch assay) and wound healing (re-closure of the scratch) was followed by time laps microcopy. To eliminate cell proliferation, these experiments were performed in serum free media. Figure 5B shows representative images of the wound healing process taken in intervals of 6 hrs. In control (scrambled) cells, the gap was almost completely closed 12 hrs after setting the defect (yellow arrowheads indicating wound edges). In contrast, when Ano1 was knocked down the defect was still clearly visible after 24 hrs. Because knockdown of Ano1 largely delayed wound healing, these results strongly support the concept of Ano1 as an important factor for cell migration [7], [32] (Figure 5C). Cell migration was also assessed using the xCELLigence system (c.f. Methods). BHY cells (expressing high levels of Ano1) migrated much faster than CAL-33 cells (expressing low levels of Ano1) (Figure 5D). Moreover, inhibition of Ano1 by AO1 strongly reduced migration of BHY cells. These results clearly indicate a role of Ano1 for cell migration. Correspondingly, lymph node metastases were more common observed in patients with Ano1 protein expressing than in Ano1 negative HNSCCs (Table S6).

### Ano1 Supports Regulatory Volume Decrease

The process of cell migration comprises cell swelling at the leading edge (front part, lamellipodium) and subsequent cell shrinkage at the rear part of the cell [33]. Increase in intracellular Ca^2+^ is essential for cell shrinkage as it activates Ca^2+^-dependent K^+^ and Cl^−^ channels at the rear part of the cell [32]. The present experiments and previous results suggest that anoctamins contribute to cell volume regulation [30], [34]. We therefore measured the impact of Ano1 on cell volume regulation by loading the cells with the fluorescence dye calcein. Reduction of calcein fluorescence indicates cell swelling (due to hypotonic bath solution; Figure 6C), while regulatory cell shrinkage (RVD) leads to a re-increase of fluorescence in the presence of hypotonic solution. We found that siRNA-knockdown of Ano1 had no effect on ATP induced cell shrinkage in either BHY or CAL-33 cells. We further examined whether anoctamins contribute to regulatory volume decrease after hypotonic swelling, as demonstrated earlier (Figure 6A,B) [30]. Surprisingly hypotonic bath solution did not induce cell swelling in BHY cells (left blue curve in Figure 6C), while knockdown of Ano1 (left red curve in Figure 6C) induced swelling with subsequent RVD (Figure 6C). These results suggest immediate and pronounced volume regulation in BHY cells, so that cell swelling does not take place in BHY cells. In contrast, CAL-33 cells, which express much lower levels of Ano1, exhibit hypotonic cell swelling (right blue curve in Figure 6C) that was augmented after knockdown of Ano1 (right red curve in Figure 6C). Taken together these results confirm the role of Ano1 for volume regulation and migration of HNSCC cells. We propose that overexpression of Ano1 in HNSCC facilitates volume regulation, which enables cell migration, thereby increasing malignancy.

## Discussion

### Ano1 as a Marker of Poor Prognosis in HNSCC and other Types of Tumors

The present report provides, for the first time, a detailed analysis of Ano1-protein expression in HNSCC and 80 different tumor types, as well as 76 types of normal tissues. In accordance with earlier studies, we found that Ano1 is rarely expressed in normal tissues [27], [34]–[36]. In HNSCC, we detected Ano1 expression in 4–19% of the analyzed samples. This is by far lower than the previously reported rates of up to 84% that were, however, based on RNA expression [37], [38]. The monoclonal Ano1 antibody used in this study is highly specific and sensitive and is thus used in diagnostic routine. However Ano1-positivity strongly correlated with adverse prognosis in a subgroup of HNSCC patients. The marker retained its significance in multivariate analysis, suggesting Ano1 expression as an independent prognostic marker for HNSCC. This did not hold true for amplification of *Ano1* or *CCND1* genes, despite their clear significance in univariate analysis. Co-amplification of *Ano1* or *CCND1* was expected, since they are separated by only 500 kb.

The 11q13 amplicon has a complex structure with up to four cores, covering many genes involved in cancer (for example *CCND1* and *EMS1*). As previously described, prevalence of the 11q13 amplification differed markedly between the different primary sites of HNSCC [39]. The fact that two thirds of Ano1 expressing HNSCC demonstrated multiple copies of 11q13, suggests genomic amplification as an important mechanism for Ano1 expression in HNSCC. Conversely, one third of HNSCC with 11q13 amplification showed expression of Ano1 protein. Taken together, Ano1 expression, rather than genomic amplification, may be used as the diagnostic standard in HNSCC. Apart from HNSCC Ano1 may be also relevant in other types of tumors. Expression of Ano1 has been described in GIST, leiomyosarcoma, and squamous cell carcinoma of head and neck or the esophagus [1], [2]. This list may be extended by breast and urinary bladder carcinoma, which demonstrate amplification of the 11q13 locus. The results suggest that Ano1 may be a driver gene within the 11q13 amplicon not only in HNSCC but also in other types of carcinoma.

### Role of Ano1 in HNSCC

The cellular function of Ano1 protein has not been fully explored. We have reported the role of Ano1 for Ca^2+^ dependent Cl^−^ secretion in epithelia [27], [34], [36]. Ayoub et al demonstrated nicely that overexpression of Ano1 stimulates cellular attachment, spreading, detachment and invasion, but not proliferation [7]. In contrast, using a Xenograft model and cell lines, Duvvuri et al detected a clear pro-proliferative effect of Ano1, which obviously occurs through Erk1/2 and activation of cyclin D1 [18]. However, we detected enhanced cellular motility and migration by Ano1, which are essential factors promoting metastasis. There is substantial evidence that cell migration and invasion is facilitated by the function of ion channels and transporters. A model has emerged in which K^+^ and Cl^−^ channels are the relevant ion channels in charge of volume regulation, thereby leading to changes in cellular shape and volume [19]. Along this line the oscillating activity of Ca^2+^ sensitive K^+^ channels has been shown to be a pre-requisite for cell migration [40]. The same class of K^+^ channels also plays a central role in cell volume regulation. During regulation of the cell volume, K^+^ channels co-operate with volume regulated anion channels (VRAC), whose molecular nature is still elusive.

We found recently that several members of the anoctamin family, including Ano1 are activated during hypoosmotic cell swelling [30]. The ability of epithelial cells to activate a Cl^−^ conductance upon cell swelling and to decrease their cell volume (regulatory volume decrease) was dependent on anoctamin proteins. Moreover, activation of these swelling activated Cl^−^ currents was reduced in the colonic epithelium and in salivary acinar cells from mice lacking expression of Ano1. While these experiments don’t claim an identity of anoctamins with VRAC, the present data again demonstrate the importance of Ano1 for volume regulation in HNSCC cells. When expression of Ano1 is inhibited, volume regulation (RVD) is reduced. The data shown in Figure 5B indicate that inhibition of Ano1 expression largely affects migration. The results also support the concept of intracellular Ca^2+^ signaling and Ca^2+^ dependent K^+^ and Cl^−^ channels being essential factors that determine cell migration and the ability to metastasize [32], [36]. We propose that Ano1 takes part in cell volume regulation and thereby supports cell migration which facilitates the emergence of metastasis.

## Materials and Methods

### Ethics Statement

In this study, sections from tissue microarrays (TMA) have been used. TMAs from archived tissue specimens were constructed and used upon approval by the Ethics Committee of Basel (Ethikkommission beider Basel, EKBB, www.ekbb.ch) on November 3^rd^, 2003. According to the approval, a written informed consent by the patients was not needed due to the retrospective nature of the study.

### Tissue Microarrays (TMA)

TMAs were constructed as described before [41]. The use of HNSCC TMA with 365, the multi-tumor TMA with 3417 and the normal TMA with 608 patient samples have been previously described [3], [22], [42].

### Fluorescence In-situ Hybridization (FISH)

For Ano1 analysis, a digoxigenated FISH probe from two BAC clones (RP11-203N8 and RP11-109F24, imaGenes GmbH, Berlin, Germany) were created as described previously [43]. For CCND1 analysis, a Spectrum Orange-labeled *CCND1* probe was used (Vysis, Downers Grove, IL). CEP11 centromeric probes were used as reference (Vysis). Indirect labeling of the digoxigenated *Ano1* FISH probe was carried out according to the ‘Fluorescent Antibody Enhancer Set for DIG Detection’ (Roche Applied Science, Rotkreuz, Switzerland). FISH signals were scored with a Zeiss fluorescence microscope and evaluated independently by CR, SS, and FR. *Ano1* and *CCND1* were defined as amplified when signals were at least twice as common as centromere 11.

### Immunohistochemistry (IHC)

For IHC, a monoclonal antibody (SP31, Cell Marque, CA) was used according to the recommended protocol and was established for the diagnostic use in GISTs. The IHC was scored using a composite scoring system: Briefly, scores were calculated by multiplying the intensity (integer between 0 and 3) with the percentage of tumor cells having this intensity. For the statistical analysis, samples with a score >0 were classified as IHC positive.

### Reverse Transcriptase PCR

Total RNA was isolated from BHY, CAL-33 and CAL-27 cells using RNeasy Mini-Kit (Qiagen; Hilden, Germany). 2 µg of total RNA was reverse-transcribed in 50 µl for 1 h at 40°C using random primer and RT. 30 cycles of RT-PCR was performed using standard procedures (GoTaq DNA Polymerase, Promega), 1 µl RT and primers for anoctamis (0.5 µM, see Table S2). For semiquantitative comparison, ß-actin was co-amplified (primer 0.05 µM). Products were analyzed on ethidium bromide-stained 2% agarose gels.

### Cell Culture, siRNA and Transfection

BHY, CAL-33 and CAL-27 cell lines were grown in Opti-MEM (Gibco) supplemented with 10% (v/v) heat inactivated fetal bovine serum (FBS, Gibco) at 5% CO_2_ and 37°C. Lipofectamine™2000 Transfection Reagent (Invitrogen, Karlsruhe, Germany) was used for transfection of hAno1 siRNA (Stealth siRNA, Invitrogen, 5′-GGUUCCCAGCCUACCUCACUAACUU-3′, 5′-AAGUUAGUGAGGUAGGCUGGGAACC-3′) or Negative Control High GC (Invitrogen) according to the manufacture’s guidelines. Experiments were performed 48 h-72 h after transfection.

### Patch Clamp

Cells were grown on glass cover slips and mounted on a perfused bath on the stage of an inverted microscope (IM35, Zeiss) and kept at 37°C. The bath was perfused continuously with Ringer solution (mM: NaCl 145, KH_2_PO_4_, K_2_HPO_4_ 1.6, D-glucose 6, MgCl_2_ 1, Ca-gluconate 1.3, pH 7.4) at about 5 ml/min. Patch-clamp experiments were performed in the fast whole-cell configuration as described earlier [30].

### Western Blot

Cells were lysed with an appropriate buffer (150 mM NaCl, 50 mM Tris–HCl, 1 mM EDTA, 1% NP-40, protease inhibitor, 100 mM DTT; pH 7.4) and DNA was sheared by sonication. Samples were quantified using a Bio-rad protein assay (Biorad) and the same amount of protein (50 µg) was separated using PAGE (7,5%). Protein were transferred to PVDF membranes (Millipore), and probed overnight at 4°C with a rabbit monoclonal anti-ANO1 antibody (Novus Biologicals, Littleton, CO, USA). Blots were visualized using a secondary HRP-conjugated anti-rabbit antibody (Acris, Herford, Germany) and Super Signal® West Pico Chemiluminescent Substrate (Pierce, Rockford, IL, USA).

### Wound Healing Assay

BHY cells were plated (5×10^5^ cells) in µ-dishes (ibidi, Martinsried, Germany) and transfected with siRNA when 70% confluent. Confluent monolayers (48 h after transfection) were then transferred to a pre-warmed Dulbecco’s Modified Eagle Medium (DMEM, Gibco) containing D-Glucose (1000 mg/l), Sodium Pyruvate, HEPES (25 mM), and aphidicolin (5 µM), and wounded with a disposable 200 µl tip. Pictures were taken every 2 min for 24 h on an inverted microscope (Observer Z1; Carl Zeiss, Inc.) operated with the ZEN 2008 software, with a polychromatic illumination system VisiChrome (Visitron) at 37°C. Image processing was done using Adobe Photoshop CS4.

### Impedance based xCELLigence Migration/proliferation Assay

The xCELLigence invasion assay (Roche, Germany) is based on changes in electrical impedance at the interphase between cell and electrode as migrating cells move through a barrier [44]. These changes were directly correlated with the migrative capacity of BHY and CAL-33 cells. The technique provides an advantage over existing methods such as boyden chamber and matrigel assays, and standard proliferation assays, respectively, since the data is obtained continuously in real-time, when compared to end-point analysis in other methods. For migration assays cells were seeded at a density of 10.000 cells/well on CIM-plates-16 migration plates. For proliferation assays cells were seeded at a density of 5.000 cells/well on E16 plates.

### Measurement of Intracellular Ca^2+^ and Cell Volume

BHY and CAL-33 cells were seeded on glass cover slips and loaded with 2 µM Fura-2/AM and 0.02% Pluronic F-127 (Invitrogen, Darmstadt, Germany) in ringer solution (mmol/l: NaCl 145; KH_2_PO_4_ 0,4; K_2_HPO_4_ 1,6; Glucose 5; MgCl_2_ 1; Ca^2+^-Gluconat 1,3) for 1 h at room temperature. Fluorescence was detected in cells perfused with Ringer’s solution (8 ml/min) at 37°C using an inverted microscope (Axiovert S100, Zeiss, Germany) and a high speed polychromator system (VisiChrome, Puchheim, Germany). Fura-2 was excited at 340/380 nm, and emission was recorded between 470 and 550 nm using a CoolSnap camera (CoolSnap HQ, Visitron). For volume measurements cells were loaded with 1 µM calcein-AM (Invitrogen, Darmstadt, Germany) and 0.01% Pluronic F-127 (Invitrogen, Darmstadt, Germany) in ringer solution for 1 h at room temperature. Calcein was excited at 490 nm, and the emission was recorded between 520 and 550 nm. Cell swelling was induced by removing of 30 mM NaCl from ringer solution. The control isotonic solution was prepared by adding 60 mM mannitol. Data were analyzed using the software package Meta-Fluor (Universal imaging, USA) and Origin (OriginLab Corporation, USA).

### Materials and Statistical Analysis

All compounds used were of highest available grade of purity and were from SIGMA. Student’s t-test (for paired or unpaired samples as appropriate), Fisher’s exact tests and ANOVA were used for statistical analysis. P<0.05 was accepted as significant. Survival curves were evaluated by the Kaplan-Meier method and log-rank test.

## Supporting Information

Table S1 **Correlation between Ano1 protein expression and 11q13 amplification in non-HNSCC tumors.** IHC (Ano1) and FISH (11q13) analysis of a small TMA constructed from Ano1 positive samples from the multi tumor TMA (A) and from breast and bladder cancers with known 11q13 amplification from previous studies [23], [24] (B). Only samples with evaluable IHC and FISH status were considered for this analysis. A) None of the Ano1 protein positive non-HNSCC tumors from the multi-tumor TMA showed amplification of the 11q13 locus. B) A substantial part of 11q13 amplified bladder cancers (6/14) and breast cancers (4/11) showed Ano1 protein expression.
(XLS)

Table S2 **Primers used for RT-PCR**
(DOC)

Table S3 **Multivariate analysis (Cox Proportional Hazards) of overall survival in HNSCC.** Genomic amplification and protein expression status of Ano1 were assessed by FISH and IHC, respectively. (CI: Confidence interval).
(XLS)

Table S4 **Multi-tumor tissue microarray.** Complete listing of Ano1 expression in various tumors and normal tissues.
(XLS)

Table S5 **Normal tissue microarray.** Complete listing of Ano1 expression in normal tissues.
(XLS)

Table S6 **Analysis of pN status in HNSCC with known Ano1 status.** Distribution of lymph node status (pN0-3) in tumor samples from the HNSCC TMA (see Table 1). Samples with Ano1 protein expression or with amplification of the *ANO1* gene showed a trend towards presence of lymph node metastases. P-values calculated by Pearson’s chi-squared test.
(XLS)

File S1 **Antiproliferative effect of Ano1 in CF-PAC1 cells.** A) Effects of Ano1-knockdown on BrdU incorporation shown as % of BrdU incorporation in cells treated with scrambled RNA (red dashed line). B) Summary of BrdU incorporation after overexpression of Ano1, shown as % of BrdU incorporation in mock transfected cells (red dashed line). B) Mean ± SEM, (number of experiments). ^#^indicates significant difference (p<0.05, ANOVA).
(PDF)
