# Supplementary material for: Enhanced Expression of ANO1 in Head and Neck Squamous Cell Carcinoma Causes Cell Migration and Correlates with Poor Prognosis
Source: PLoS One. 2012 Aug 17;7(8):e43265. doi: 10.1371/journal.pone.0043265 (PMC3422276; doi:10.1371/journal.pone.0043265)
Supplement: Table S2 — Primers used for RT-PCR (DOC) [file pone.0043265.s002.doc]

**Supplementary Table 2:** Primers used for RT-PCR

| Anoctamin | Primer | Sequenz | Size (bp) |
| --- | --- | --- | --- |
| ANO1 (16a) | sense  antisense | atgagggtca acgagaagta c  ggagaaggga taggagagtc | 675 |
| ANO2 (16b) | sense  antisense | ggacaccttc tttgataatg c  gcattctgct ggtcacacat | 414 |
| ANO3 (16c) | sense  antisense | cttccctctt ccagtcaac  aaacatgata tcggggcttg | 461 |
| ANO4 (16d) | sense  antisense | gaacccatgg agcagaaaac  gcttcaaact ggggtcgtat | 496 |
| ANO5 (16e) | sense  antisense | gaatgggacc tggtggac  gagtttgtcc gagcttttcg | 713 |
| ANO6 (16f) | sense  antisense | ggagttttgg aagcgacgc  gtatttctgg attgggtctg | 325 |
| ANO7 (16g) | sense  antisense | cctcgactgc cctttctg  ggcacggtac aggatgatag a | 379 |
| ANO8 (16h) | sense  antisense | ggaggaccag ccaatcatc  tccatgtcat tgagccag | 705 |
| ANO9 (16j) | sense  antisense | gcagccagtt gatgaaatc  gctgcgtagg taggagtgc | 472 |
| ANO10 (16k) | sense  antisense | gtgaagagga aggtgcagg  tcatcgtttc aaaagccaac t | 301 |
| β-actin | Sense  antisense | caacggctcc ggcatgtg  cttgctctgg gcctcgtc | 151 bp |
