# Supplementary figures and images for: Enhanced Expression of ANO1 in Head and Neck Squamous Cell Carcinoma Causes Cell Migration and Correlates with Poor Prognosis
Source: PLoS One. 2012 Aug 17;7(8):e43265. doi: 10.1371/journal.pone.0043265 (PMC3422276; doi:10.1371/journal.pone.0043265)

A

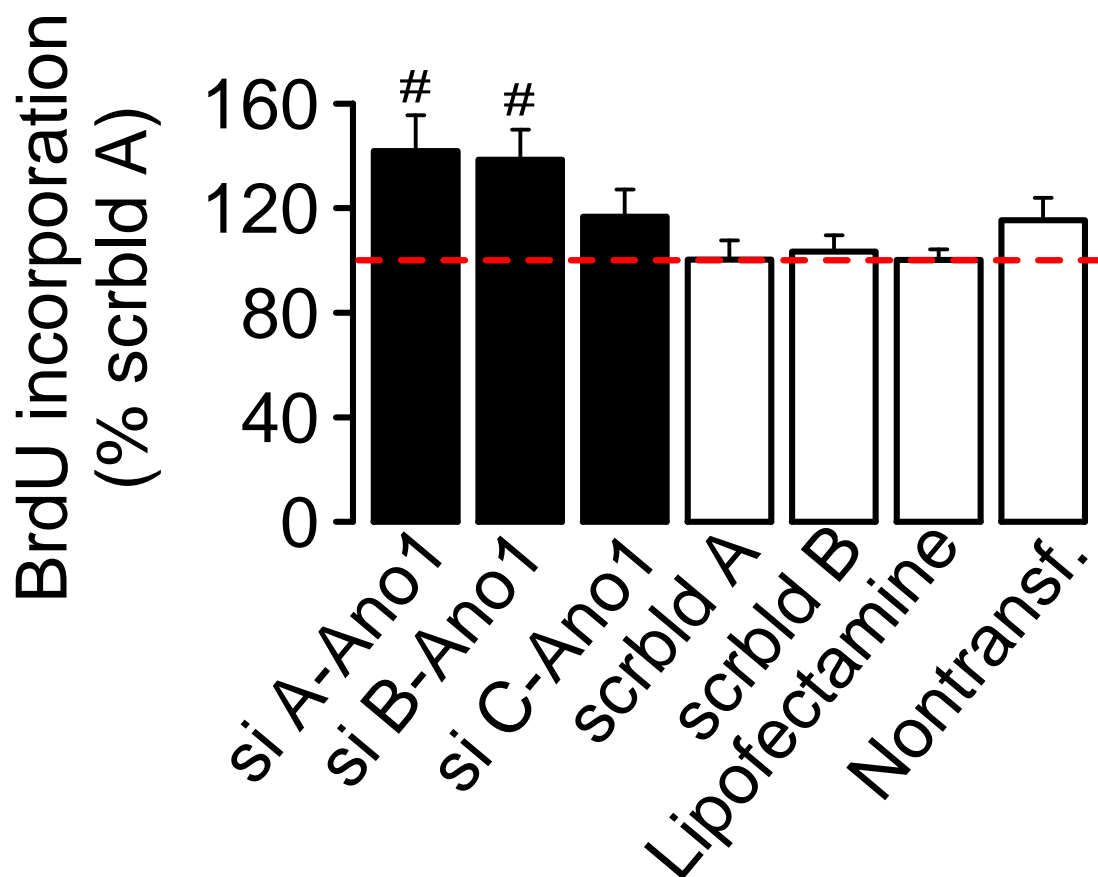

B

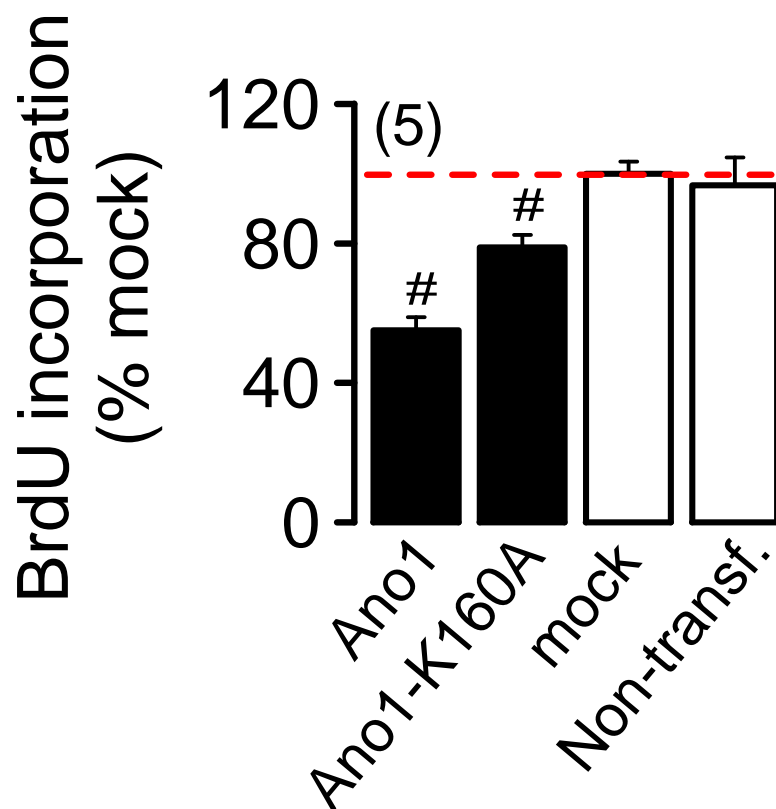

Supplement: File S1 — Antiproliferative effect of Ano1 in CF-PAC1 cells. A) Effects of Ano1-knockdown on BrdU incorporation shown as % of BrdU incorporation in cells treated with scrambled RNA (red dashed line). B) Summary of BrdU incorporation after overexpression of Ano1, shown as % of BrdU incorporation in mock transfected cells (red dashed line). B) Mean ± SEM, (number of experiments). #indicates significant difference (p<0.05, ANOVA). (PDF) [file pone.0043265.s007.pdf]
